# LoRa 2.4 GHz Communication Link and Range

**DOI:** 10.3390/s20164366

**Published:** 2020-08-05

**Authors:** Thomas Janssen, Noori BniLam, Michiel Aernouts, Rafael Berkvens, Maarten Weyn

**Affiliations:** IDLab-Faculty of Applied Engineering, University of Antwerp-imec, Sint-Pietersvliet 7, 2000 Antwerp, Belgium; thomas.janssen@uantwerpen.be (T.J.); noori.bnilam@uantwerpen.be (N.B.); michiel.aernouts@uantwerpen.be (M.A.); rafael.berkvens@uantwerpen.be (R.B.)

**Keywords:** low power wide area networks, LPWAN, LoRaWAN, LoRa, 2.4 GHz, wireless sensor networks, path loss modeling, range estimation

## Abstract

Recently, Semtech has released a Long Range (LoRa) chipset which operates at the globally available 2.4 GHz frequency band, on top of the existing sub-GHz, km-range offer, enabling hardware manufacturers to design region-independent chipsets. The SX1280 LoRa module promises an ultra-long communication range while withstanding heavy interference in this widely used band. In this paper, we first provide a mathematical description of the physical layer of LoRa in the 2.4 GHz band. Secondly, we investigate the maximum communication range of this technology in three different scenarios. Free space, indoor and urban path loss models are used to simulate the propagation of the 2.4 GHz LoRa modulated signal at different spreading factors and bandwidths. Additionally, we investigate the corresponding data rates. The results show a maximum range of 333 km in free space, 107 m in an indoor office-like environment and 867 m in an outdoor urban context. While a maximum data rate of 253.91 kbit/s can be achieved, the data rate at the longest possible range in every scenario equals 0.595 kbit/s. Due to the configurable bandwidth and lower data rates, LoRa outperforms other technologies in the 2.4 GHz band in terms of communication range. In addition, both communication and localization applications deployed in private LoRa networks can benefit from the increased bandwidth and localization accuracy of this system when compared to public sub-GHz networks.

## 1. Introduction

More than a decade ago, Long Range (LoRa) was invented to—as the name indicates—provide a low power wide area network (LPWAN) protocol operating at sub-GHz frequencies. Because of local spectrum regulations, LoRa hardware modules need to be adapted to operate in different frequency bands. For example, in the US, the 915 MHz band is used, while in Europe, most LoRa chipsets operate in the 868 MHz band. In the past, we have conducted extensive research on communication and localization with sub-GHz LPWAN.

Semtech, the founding member of the LoRa Alliance, recently released a LoRa chipset operating at 2.4 GHz. The move from sub-GHz to 2.4 GHz was mainly done in order to use the globally available 2.4 GHz Industrial, Scientific and Medical (ISM) band. This tackles the problem of having to develop multiple chipsets which operate at different frequency bands, thus paving the way for the development of a universal chipset which can operate anywhere in the world. This is especially valid for track and trace applications, where goods cross different zones worldwide. Nevertheless, the potential benefits of LoRa in the 2.4 GHz band have not yet been investigated thoroughly, which constitutes the main objective of this paper.

In general, the range of sub-GHz LPWAN varies from a few kilometers in urban environments to more than 10 km in rural environments. Obviously, the maximum communication range between the transmitter and receiver also depends on the used frequency band. The goal of this research is to study the inverse relationship between the maximum communication range and the corresponding data rate of LoRa in the 2.4 GHz band.

Since sub-GHz LoRa devices are very limited in terms of their number of possible transmissions—i.e., to comply with duty cycle regulations—they are typically used for applications that do not require frequent communication. For example, Sendra et al. proposed a LoRa-based forest fire detection system that transmits sensor measurements every 28 min [1]. Besides this, the long-range communication benefit of sub-GHz LoRa has been exploited to provide emergency services in GPS-less environments [2]. Other application examples with sub-GHz LoRa include smart meter reading, environmental monitoring, smart farming and smart building applications [3,4]. On the other hand, LoRa devices that operate at 2.4 GHz are able to transmit at higher data rates because of the higher available bandwidth [5]. Consequently, this technology can offer a balance for applications that require a higher data rate than LPWANs and a longer communication range than classic 2.4 GHz technologies such as Wi-Fi and Bluetooth. Additionally, a higher bandwidth also allows for more accurate time-based localization [6]. Thus, LoRa at 2.4 GHz represents an interesting solution for a variety of applications that involve indoor localization, such as warehouse management, but also for applications that require outdoor localization, such as construction site monitoring, livestock tracking, etc. Moreover, the adoption of this technology can add flexibility to applications that require consistent asset tracking in both indoor and harsh outdoor environments; e.g., smart ports.

The main contributions of this work are the following:

- We provide an overview of the physical layer of LoRa operating at 2.4 GHz.
- We discuss the maximum communication range and data rate in three different scenarios: free space, indoor and urban environments.
- We discuss the impact of moving from LoRa at sub-GHz bands to 2.4 GHz on communication and localization applications.

The remainder of this paper is structured as follows. Section 2 contains related work regarding LoRa in both sub-GHz bands and the 2.4 GHz band. Subsequently, a mathematical background of LoRa at 2.4 GHz is provided in Section 3. Next, three path loss models are presented in Section 4 in order to estimate the maximum communication range and corresponding data rate in a free space and in indoor and urban environments. The results of these estimations are shown in Section 5 and compared to other technologies operating in the 2.4 GHz band in Section 6, which also discusses the impact on the application potential of LoRa at 2.4 GHz. Finally, general conclusions are drawn in Section 7.

## 2. Related Work

With the aim of providing a low power, long-range wireless communication protocol, Semtech acquired the startup company Cycleo, which developed LoRa in 2009. The authors in [7] provided an in-depth analysis of the functional components of both the physical (LoRa) and data link (LoRaWAN) layer of the popular LPWAN protocol operating in unlicensed bands.

Sub-GHz LoRa-based communication has been studied thoroughly in the past decade. Acting as a layer above LoRa, LoRaWAN is widely used to communicate small messages over large areas. A LoRaWAN network can fully cover a city-scale environment with only a few gateways. The mobility of LoRaWAN has been addressed in [8]. The forwarding of an uplink message by multiple gateways ensures that a handover can take place without any loss of data. In another study, a robust frame detection algorithm was proposed in order to detect LoRa-modulated frames with minimal complexity implementations [9]. Furthermore, a frame relay strategy was found to be a feasible way to improve the link quality of poorly connected nodes and successfully extend the range of LoRaWAN [10].

In addition to the communication aspect of LPWAN, a significant amount of research, both from academic and industrial perspectives, has involved the location objects with of sub-GHz LPWAN. We conducted extensive research on localization with different LPWAN technologies. We evaluated the accuracy of received signal strength (RSS)-based localization algorithms for Sigfox [11], LoRaWAN [12] and NB-IoT [13]. We also provided an angle of arrival (AoA) estimation solution that is suitable for use in sub-GHz bands [14,15]. Furthermore, we devised a probabilistic localization model that combines time difference of arrival (TDOA) and AoA estimations in a sub-GHz LoRa network [6].

Recently, Semtech released the SX1280 chipset, which operates at 2.4 GHz [5]. In an application note, the company provides an introduction to time-of-flight (ToF) ranging possibilities with the radio chip [16]. A proof-of-concept implementation for coherent multi-channel ranging with this LoRa radio chip is provided in [17].

A few concerns are raised by the move from sub-GHz frequencies to the widely used unlicensed 2.4 GHz ISM band. The interference properties of wireless local area networks (WLAN, also denoted as Wi-Fi) devices operating in this band were characterized in [18]. In another study, coexistence issues with Wi-Fi and LoRa 2.4 GHz were addressed [19]. The theoretical assumptions about the high robustness of the system against interference are confirmed. However, it was found that the robustness highly depends on both the configuration of LoRa and the properties of interfering technologies. Moreover, Polak et al. used different bandwidths in their evaluation and compared them to the bandwidths that are currently available for LoRa at 2.4 GHz.

The maximum communication range in the 2.4 GHz band strongly depends on the technology and its respective transmission power. For the latest Bluetooth Low Energy (BLE 5) standard, the maximum communication ranges in different scenarios are summarized in [20]. Typical ranges are 50 m in an indoor scenario, 165 m in an outdoor non-line-of-sight (NLoS) scenario and 780 m in a LoS scenario. In this research, we will investigate the range of 2.4 GHz LoRa in a free space and in indoor and outdoor scenarios.

## 3. LoRa in the 2.4 GHz Band

The physical layer of LoRa is a proprietary and closed source. Therefore, there are no official references or protocol specifications for the transmitted RF signal [21]. Accordingly, several research groups have been working to provide an understanding of the LoRa modulation scheme in the sub-GHz frequency band. Vangelista [22], for instance, has provided a mathematical model, called Frequency Shift Chirp Modulation (FSCM), that describes the LoRa modulation process. The same model has been adopted by Bernier et al. [9]. On the other hand, Knight [21], Robyns et al. [23] and Ghanaatian et al. [24] have provided a model called Chirp Spread Spectrum (CSS) modulation based on the reverse engineering of LoRa’s physical layer. Even though the formulation of the LoRa modulation scheme in the literature has been provided for the sub-GHz frequency band, the basic response of the modulation is expected to be the same for LoRa signals in the 2.4 GHz frequency band. Therefore, in this section, we modify the available physical layer models of sub-GHz LoRa to make them suitable for use in the 2.4 GHz frequency band.

Assume $x_{s}\left(k\right)$ is the transmitted LoRa sample; then, the received sampled signal $x_{r}\left(k\right)$ with index *k* can be expressed as

(1) $$x_{r}\left(k\right)=a_{r}x_{s}\left(k-\tau \right)e^{i2\pi \Delta fk}+\omega \left(k\right),$$

where $a_{r}<1$ is the received signal amplitude, τ is the time delay of the sample $x_{s}\left(k\right)$ at the receiver, Δf is the frequency offset between the transmitter and the receiver, and ω(k) is the identically independently distributed (i.i.d.) complex-valued Gaussian noise with zero-mean and variance $\sigma^{2}$; i.e., $CN(0,\sigma^{2})$. The time and frequency synchronization are beyond the scope of this paper. Therefore, in the following, we will consider a simplified version of (1), shown in (2).

(2) $$x_{r}\left(k\right)=a_{r}x_{s}\left(k\right)+\omega \left(k\right)$$

The LoRa standard linear upchirp—also called a base chirp—can be expressed as [23,24]

(3) $$x_{s}\left(k\right)=e^{i2\pi \left(\frac{\mathrm{BW}}{2K}k^{2}+f_{\circ }k\right)},$$

where BW is the operational bandwidth of the LoRa signal in the 2.4 GHz frequency band (as shown in Table 1) and $K=2^{\mathrm{SF}}/\mathrm{BW}$ is the symbol duration, with SF representing the spreading factor (also shown in Table 1). Finally, $f_{\circ }$ is the initial frequency, which can be expressed as

(4) $$f_{\circ }=s\frac{\mathrm{BW}}{2^{\mathrm{SF}}},$$

where $s\in \left\{0,1\ldots 2^{\mathrm{SF}}\right\}$ is the transmitted data symbol. Setting s=0 results in an upchirp, in which the frequency continuously increases during the symbol duration *K*.

We can also present (3) as

(5) $$x_{s}\left(k\right)=W_{K}^{\frac{\mathrm{BW}}{2}k^{2}+Kf_{\circ }k},\;\;\;\;W_{K}=e^{i2\pi /K}.$$

The model in (5) is the linearly cyclically shifted version of a base Zadoff–Chu (ZC) sequence [25]. The ZC sequence possesses a unique autocorrelation property, in which the periodic autocorrelation is orthogonal (i.e., equal to zero) for all shifted replicas [26]. Therefore, the LoRa communication protocol uses this unique property to impose a random multiple access technique. Accordingly, an efficient utilization of the unlicensed spectrum can be obtained. The correlation between the received signal and the base chirp leads to

(6) $$\begin{array}{cc} z(k) & =\frac{1}{K}\sum_{p=0}^{K-1}x\left(k+p\right)x_{s}^{*}{\left(k\right)}_{\mathrm{mod}\;K}\\ \; & =\frac{1}{K}\sum_{p=0}^{K-1}\left(a_{r}x_{s}\left(k+p\right)+\omega \left(k+p\right)\right)x_{s}^{*}\left(k\right)\\ \; & =\left\{ \begin{array}{cc} a_{r}E_{s}+\nu_{\omega } & \mathrm{for}\;p=0\\ \nu_{\omega } & \mathrm{for}\;p\neq 0 \end{array}\right. \end{array},$$

where $E_{s}$ is the energy of the symbol $x_{s}$. Furthermore, $\nu_{\omega }$ is the correlation between complex noise and the base chirp, which can be expressed as

(7) $$\nu_{\omega }=\frac{1}{K}\sum_{p=0}^{K-1}x_{s}^{*}\left(k\right)\omega \left(k+p\right),$$

in which $\nu_{\omega }∼CN\left(0,\sigma^{2}/K\right)$.

**Figure 1:**
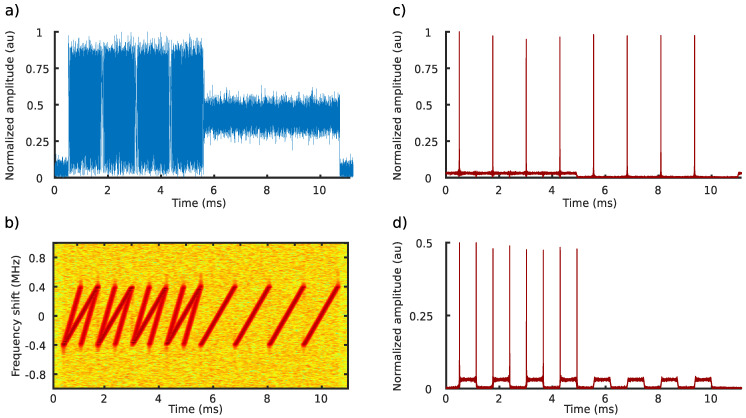
Two received Long Range (LoRa) signals constitute eight preamble upchirp symbols at 2.4 GHz with a bandwidth equal to 812 kHz. The spreading factor (SF) of the short signal, which ended after approximately 5 ms, is equal to 9, while the SF of the long-duration signal is equal to 10. Figures (**a**) and (**b**) represent the combined received signals in the time domain and in the spectrogram (i.e., time and frequency) domain, respectively. Figures (**c**) and (**d**) are the cross-correlation functions (6) when the received signals have been cross-correlated with base chirps of the SF equal to 10 and 9, respectively.

Figure 1 presents two received LoRa signals that constitute eight preamble (upchirp) symbols at 2.4 GHz with a bandwidth equal to 812 kHz. The short signal was transmitted at an SF of 9, while the longer signal was transmitted at an SF of 10. Figure 1a,b shows the combined received signals in the time domain and in the spectrogram (i.e., time and frequency) domain, respectively. Figure 1c,d shows the cross-correlation functions (6) when the received signals have been cross-correlated with base chirps of the SF equal to 10 and 9, respectively. It is clear that the two signals can be distinguished correctly, even though they interfere with each other. The unique orthogonality property of the ZC sequence allows the LoRa communication system to provide a multiple access technique in the 2.4 GHz frequency band.

## 4. Path Loss Modeling

In order to obtain the maximum communication range of a LoRa signal at 2.4 GHz (further denoted as *d*), we need to find the maximum link budget for which the signal can be received properly; i.e., at the receiver sensitivity $P_{RX}$. This receiver sensitivity depends on two key factors: the used spreading factor (SF) and bandwidth (BW). While the SF can range from 5 to 12, the possible bandwidths of LoRa at 2.4 GHz are 203, 406, 812 and 1625 kHz. Furthermore, the combination of a certain SF and BW results in a certain data rate, along with the receiver sensitivity, as shown in Table 2. The raw data rate $R_{b}$, expressed in kbit/s, can be calculated as

(8) $$R_{b}=\frac{SF*BW}{2^{SF}},$$

with SF and BW as defined in Table 1. As an example, a LoRa signal transmitted with an SF of 8 and a BW of 406 kHz results in a receiver sensitivity of −116 dBm and a data rate of 12.69 kbit/s. The receiver sensitivities and data rates used in this work originate from the datasheet of the Semtech SX1280 LoRa module [5].

The total link budget of a wireless communication signal propagating from the transmitter to receiver can be represented as [27]

(9) $$P_{RX}=P_{TX}+G_{TX}-L_{TX}-L_{p}\left(d\right)+G_{RX}-L_{RX},$$

where $P_{RX}$ is the received power in dBm, $P_{TX}$ is the transmission power in dBm, $G_{TX}$ is the antenna gain at the transmitter in dBi, $L_{TX}$ is the cable loss in dB, $L_{p}\left(d\right)$ is the path loss in dB in terms of the function from the distance *d*, $G_{RX}$ is the antenna gain at the receiver in dB and $L_{RX}$ is the cable loss at the receiver in dB. Except for the path loss, all these parameters are set to typical values which are commonly used when simulating a wireless communication link between two dipole antennas [27]. The values of these parameters are summarized in Table 1.

The path loss $L_{p}\left(d\right)$ is defined as the propagation loss caused by the signal traveling from the transmitter to receiver over a distance *d*. The goal in this research is to maximize *d* while still being able to successfully receive the LoRa-modulated signal at the receiver. For the sake of simplicity, the simulated LoRa signal contains eight preamble symbols and no payload bytes.

Depending on the environment, the path loss should be modeled differently. Therefore, in the next three subsections, we discuss indoor, outdoor (urban) and free space path loss models to translate the propagation loss into a distance between the transmitter and receiver. The parameters required by these models are also summarized in Table 1. No fade margin is taken into account; this is further discussed in Section 6.

### 4.1. Free Space Environment

The first scenario can be described as a free space environment in which there is a line of sight (LoS, i.e., the primary Fresnel zone is to be at least 60% clear.) between the TX and RX locations. In this case, we can use the widely used Free Space Path Loss (FPSL) model to evaluate the maximum communication range. This model calculates the loss between two isotropic radiators in free space, without considering any obstacles, reflections or interference. The model solely relies on the frequency and distance between the transmitter and receiver to calculate the path loss:

(10) $$L_{p,LoS}\left(d\right)=32.44+20\log_{10}\left(f\right)+20\log_{10}\left(d\right),$$

where *f* is in MHz and *d* is in km. The combination of a given SF and BW yields a certain sensitivity $P_{RX}$. Consequently, given the maximum path loss obtained from (9), we can calculate the maximum distance as

(11) $$d=10^{(L_{p,LoS}\left(d\right)-32.44-20\log_{10}\left(f\right))/20}.$$

### 4.2. Indoor Environment

In the second scenario, we evaluate the maximum communication range in an indoor environment. To this end, we slightly adapt a heuristic algorithm that was developed based on real measurements in an office-like environment [28]. The path loss model is based on the Indoor Dominant Path (IDP) model, which focuses on the dominant path between the TX and RX location. In general, the total path loss is the sum of the distance loss, accumulated wall loss and interaction loss and can be calculated as

(12) $$L_{p,in}\left(d\right)=L_{p_{0}}\left(d_{0}\right)+10*n*\log_{10}\left(\frac{d}{d_{0}}\right)+\sum_{i}L_{W_{i}}+\sum_{j}L_{B_{j}},$$

where $L_{p_{0}}\left(d_{0}\right)$ represents the path loss at a distance $d_{0}$ and *n* is the path loss exponent. The accumulated wall loss is the sum of losses $L_{W_{i}}$ caused by each wall along the dominant path. Finally, the interaction loss is the sum of losses $L_{B_{j}}$ caused by all directional changes of the propagating signal.

Given the semi-empirical nature of this path loss model, some parameters need to be set to commonly used values in order to provide a generally applicable model that can predict ranges in other indoor environments. Therefore, $L_{p_{0}}\left(d_{0}\right)$ is set to 40 dB at a distance $d_{0}=1$ m, as suggested in [28]. The path loss exponent is set to n=5, which is generally used for obstructed paths inside buildings [29]. For the accumulated wall and interaction loss, values of 6 and 3 dB have been taken into account, as found specifically for the office-like environment in [28]. Consequently, the path loss model can be simplified to

(13) $$L_{p,in}\left(d\right)=40+5*10*\log_{10}\left(d\right)+6+3.$$

Thus, the range can be empirically estimated based on the path loss:

(14) $$d=10^{(L_{p,in}\left(d\right)-49)/50}.$$

### 4.3. Urban Environment

An urban path loss model is used in the third scenario to evaluate the range of LoRa at 2.4 GHz in an outdoor city-scale environment. The Okumura–Hata Urban Path Loss model is an empirical model that is often used in sub-GHz wireless communication systems. While the COST-231 urban model extended its use up to 2 GHz, the Electronic Communication Committee (ECC) modified the original Okumura–Hata model to work with frequencies up to (and beyond) 3 GHz in the ECC-33 model [30,31]. Therefore, the ECC-33 model is suitable to evaluate the maximum communication range of LoRa at 2.4 GHz in an urban environment. The path loss equation for this model is given by

(15) $$L_{p,urban}\left(d\right)=A_{fs}+A_{bm}+G_{b}+G_{m},$$

where $A_{fs}$ is the free space attenuation, $A_{bm}$ is the basic median path loss, $G_{b}$ is the base station height gain factor and $G_{r}$ is the receiver height gain factor, which can be calculated as

(16) $$A_{fs}=92.4+20\log_{10}\left(d\right)+20\log_{10}\left(f\right),$$

(17) $$\begin{array}{ccc} A_{bm} & = & 20.41+9.83\log_{10}\left(d\right)+7.894log_{10}\left(f\right)\\ \; & \; & +\;9.56{\left[\log_{10}\left(f\right)\right]}^{2}, \end{array}$$

(18) $$\begin{array}{ccc} G_{b} & = & \log_{10}\left(\frac{h_{b}}{200}\right)\left\{13.958+5.8{\left[\log_{10}\left(d\right)\right]}^{2}\right\},\;\mathrm{and} \end{array}$$

(19) $$\begin{array}{ccc} G_{m} & = & \left[42.57+13.7\log_{10}f\right]\left[\log_{10}\left(h_{m}\right)-0.585\right] \end{array}$$

for medium-sized urban environments. Given the complexity of this set of equations, we extract the maximum range by iterating over values of *d* from 1 m to 10 km and solving the optimization problem given a certain path loss $L_{p,urban}\left(d\right)$.

## 5. Range Versus Data Rate: Results

**Figure 2:**
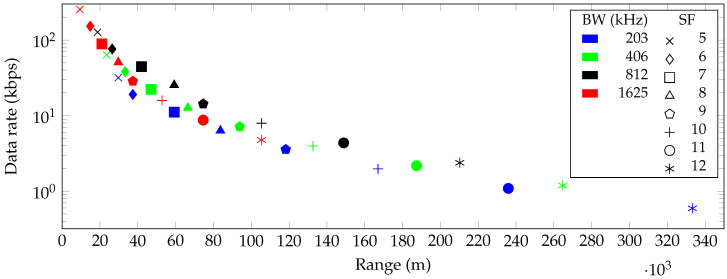
Communication range and data rate for every combination of spreading factor (SF) and bandwidth (BW) in a free space line of sight (LoS) environment.

**Figure 3:**
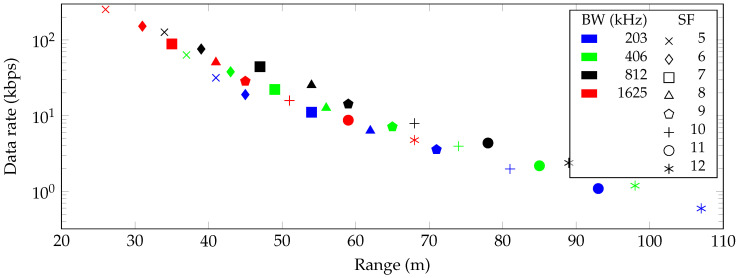
Communication range and data rate for every combination of spreading factor (SF) and bandwidth (BW) in an indoor environment.

**Figure 4:**
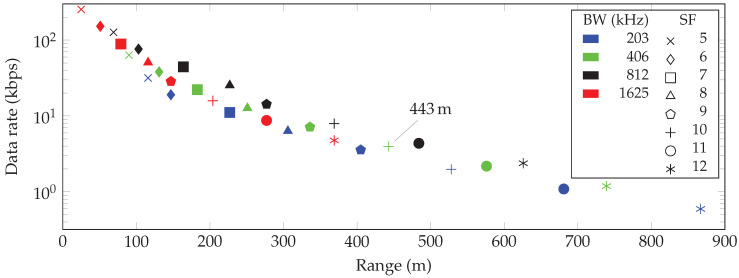
Communication range and data rate for every combination of spreading factor (SF) and bandwidth (BW) in an urban environment.

Figure 2, Figure 3 and Figure 4 show the maximum communication range and corresponding data rate at each combination of SF and bandwidth for the free space and indoor and urban environments, respectively. In all cases, the highest possible data rate decreases in a logarithmic way when the communication range between the transmitter and receiver increases.

Using the Free Space Path Loss model, it is found that a 2.4 GHz LoRa signal can travel up to 333 km in free space and still be received properly. Obviously, this is only a theoretical range and cannot be realized in real-world environments.

The performance of a more realistic indoor path loss model has been visualized in Figure 3. When transmitting with a spreading factor of 12 and the lowest bandwidth (i.e., 203 kHz), the path loss equals 150.5 dB. Consequently, a maximum communication range of 107 m can be achieved. Furthermore, the highest possible data rate at that range becomes 0.595 kbit/s. At the other extreme, the highest achievable data rate of 253.91 kbit/s is possible at a range of up to 26 m.

Finally, the communication range of the urban ECC-33 path loss model varies from 25 m at the highest achievable data rate of 253.91 kbit/s to 867 m at the lowest possible data rate of 0.595 kbit/s.

## 6. Discussion

We investigated the maximum communication range of LoRa in the 2.4 GHz band, which is defined as the maximum distance between a transmitter and receiver at which a LoRa-modulated message can be received properly. Our investigations included three environments: LoS free space, NLoS indoor and urban outdoor. In all three scenarios, we maximized the range by reducing the bandwidth and increasing the spreading factor.

Based on the total link budget of the wireless communication system, including receiver sensitivity, antenna gains and cable losses, we were able to estimate the range of LoRa at 2.4 GHz. It is important to note that we did not include a fade margin in the link budget calculations. The fade margin can be defined as the level of received power in excess of that required for a specified minimum level of system performance. The reason for excluding this loss parameter in (9) is the high variability of fade margin in different scenarios. For instance, a 5 dB fade margin decreases the maximum urban range from 867 m to 576 m, while a 10 dB fade margin further decreases the range to 369 m. Thus, this should be taken into account when analyzing the results. Nonetheless, the largest factor by far in a link budget is the path loss.

The free space line-of-sight scenario resulted in a theoretical maximum range of 333 km when transmitting at the highest SF and using the lowest bandwidth. In reality, the signal will always have to cope with obstacles, multipath propagation effects and interference with other signals. Therefore, these ranges will never be achieved in a real-world environment. Nevertheless, the results of the Free Space Path Loss model are useful as a benchmark as they enable us to compare them with different frequencies and technologies. For instance, the maximum range of LoRa at 868 MHz calculated with the FSPL model equals 921 km, which is almost three times the range of LoRa at 2.4 GHz.

For the indoor range estimation of 2.4 GHz LoRa, an indoor path loss model was evaluated. In order to provide the highest possible accuracy, a model based on real-world measurements was adopted and slightly modified, taking into account both wall and interaction loss [28]. The estimated range varies from 26 m to 107 m, depending on the SF and BW. It should be noted that a path loss exponent of 5 was chosen, simulating an obstructed indoor environment. However, in an indoor LoS scenario, the range might therefore be increased.

Finally, the maximum communication range in an urban environment was found to be 867 m. As indicated in Figure 4, the range at an SF of 10 and a BW equal to 406 kHz is 443 m. This is similar to the results of the experiments with the SX1280 chipset carried out by Wolf et al. [17]. They found that ToF ranging with the aforementioned SF and BW failed for ranges over about 500 m. Although they only investigated the ranging feature at an SF of 10 and a BW equal to 406 kbit/s and 1625 kbit/s, this partially validates our range estimations of the ECC-33 path loss model.

Besides the communication range, we investigated the data rates for all combinations of SFs and BWs and consequently associated this information with the highest achievable range. The highest possible data rate of LoRa at 2.4 GHz equals 253.91 kbit/s, which is almost seven times higher than the maximum data rate of LoRa at 868 MHz. This data rate can be achieved if the distance between the transmitter and receiver is not greater than 9393 m, 26 m and 25 m in a free space, indoor and urban environment, respectively.

Some significant differences in terms of range arise when comparing LoRa to other technologies operating in the 2.4 GHz band. As mentioned earlier, the range of the latest Bluetooth standard equals 50 m and 165 m in an indoor and outdoor environment, respectively. Moreover, the maximum range of 2.4 GHz Wi-Fi networks typically varies around 100 m. Thus, the outdoor range of LoRa is more than five times larger than the outdoor range of BLE 5 and more than eight times larger compared to typical IEEE 802.11 networks. This is mainly due to the lower bandwidth and data rates used in LoRa, as well as the robustness of the LoRa-modulated signal. These numbers clearly indicate the significant difference in intended applications between LoRa (such as long-range communication and localization) and Wi-Fi and Bluetooth (such as video and audio streaming).

Since LoRa modulation at 2.4 GHz has a higher bandwidth than LoRa modulation at 868 MHz, the rising edge of a signal pulse can be determined more accurately. Therefore, we expect that time-based localization methods for this technology will result in lower estimation errors. However, the results in this paper show that it is not possible to achieve the same long communication ranges as LoRa at 868 MHz and with other sub-GHz LPWANs. Therefore, more LoRa receivers have to be deployed to cover wide areas, which makes it a less feasible solution to build large public networks. On the other hand, 2.4 GHz LoRa is an interesting option for both communication and localization in privately deployed networks that are purposed for asset tracking and monitoring in large warehouses, construction sites, farms, etc.

## 7. Conclusions

With the move from sub-GHz frequency bands to the globally available 2.4 GHz ISM band, hardware manufacturers are able to design a uniform LoRa chipset which functions independently of the region of deployment. However, as a consequence of moving to a higher frequency, the range of LoRa is reduced when compared to the sub-GHz range of several kilometers. In this paper, we first provide an overview of LoRa operating in the 2.4 GHz band. By calculating the link budget of a chipset operating in this band, the range of a LoRa modulated signal is estimated in a free space and in indoor and outdoor scenarios. When compared to other technologies operating in the 2.4 GHz band, LoRa outperforms them in terms of communication range, due to the configurable SF and bandwidth. Thus, when configuring a LoRa channel at 2.4 GHz, a trade-off between range and data rate should be taken into account. Moreover, this trade-off leads to more flexible applications, such as the localization of assets in a private LoRaWAN network, which will be investigated in future work.

## Figures and Tables

**Table 1:** Parameters used for path loss modeling.

Model Parameter	Symbol	Value	Unit
Frequency	*f*	2.4	GHz
Spreading factor	SF	5–12	-
Bandwidth	BW	203/406/812/1625	kHz
Code rate	$R_{C}$	4/5	-
Transmission power	$P_{TX}$	12.5	dBm
Transmitter antenna gain	$G_{TX}$	2	dBi
Transmitter cable losses	$L_{TX}$	−2	dB
Fading margin	$L_{m}$	0	dB
Receiver antenna gain	$G_{RX}$	2	dBi
Receiver cable losses	$L_{RX}$	−2	dB
Base station height	$h_{b}$	20	m
Mobile station height	$h_{m}$	2	m

**Table 2:** Receiver sensitivities ($P_{RX}$ in dBm) and corresponding data rates ($R_{D}$ in ) of the SX1280 LoRa module for every combination of spreading factors (SFs) and bandwidths (BWs).

	BW (kHz)							
	203		406		812		1625	
SF	$P_{RX}$	$R_{D}$	$P_{RX}$	$R_{D}$	$P_{RX}$	$R_{D}$	$P_{RX}$	$R_{D}$
5	−109	31.72	−107	63.44	−105	126.88	−99	253.91
6	−111	19.03	−110	38.06	−108	76.13	−103	152.34
7	−115	11.1	−113	22.2	−112	44.41	−106	88.87
8	−118	6.34	−116	12.69	−115	25.38	−109	50.78
9	−121	3.57	−119	7.14	−117	14.27	−111	28.56
10	−124	1.98	−122	3.96	−120	7.93	−114	15.87
11	−127	1.09	−125	2.18	−123	4.36	−117	8.73
12	−130	0.595	−128	1.19	−126	2.38	−120	4.76
