# Correction: Janssen, T., et al. LoRa 2.4 GHz Communication Link and Range. *Sensors* 2020, *20*, 4366

**DOI:** 10.3390/s20226428

**Published:** 2020-11-10

**Authors:** Thomas Janssen, Noori BniLam, Michiel Aernouts, Rafael Berkvens, Maarten Weyn

**Affiliations:** IDLab-Faculty of Applied Engineering, University of Antwerp-imec, Sint-Pietersvliet 7, 2000 Antwerp, Belgium; thomas.janssen@uantwerpen.be (T.J.); noori.bnilam@uantwerpen.be (N.B.); michiel.aernouts@uantwerpen.be (M.A.); rafael.berkvens@uantwerpen.be (R.B.)

The authors wish to make the following corrections to this paper [1]:

The authors discovered a numerical error in the paper, which impacts part of the results in the paper. More specifically, the authors subtracted a negative loss in the link budget calculations, which over-estimated the resulting ranges in the original Figure 2, Figure 3 and Figure 4. However, the discussion and conclusions of the published paper remain the same. Thus, the authors adapted the numerical values in the results accordingly and updated Table 1 and Figure 2, Figure 3 and Figure 4.

## Changes in Abstract

In line 10, the sentences “The results show a maximum range of 333 km in free space, 107 m in an indoor office-like environment and 867 m in an outdoor urban context.” should be changed to “The results show a maximum range of 133 km in free space, 74 m in an indoor office-like environment and 443 m in an outdoor urban context.”

## Changes in Section 3. LoRa in the 2.4 GHz Band

In the original article, there was a mistake in Table 1 as published. The Value of Model Parameter “Transmitter cable losses” and “Receiver cable losses” in Table 1 should be changed from −2, −2 to 2, 2. The corrected Table 1 appears below. 

**Table 1:** Parameters used for path loss modeling.

Model Parameter	Symbol	Value	Unit
Frequency	*f*	2.4	GHz
Spreading factor	*SF*	5–12	–
Bandwidth	*BW*	203/406/812/1625	kHz
Code rate	*R_C_*	4/5	–
Transmission power	*P_TX_*	12.5	dBm
Transmitter antenna gain	*G_TX_*	2	dBi
Transmitter cable losses	*L_TX_*	2	dB
Fading margin	*L_m_*	0	dB
Receiver antenna gain	*G_RX_*	2	dBi
Receiver cable losses	*L_RX_*	2	dB
Base station height	*h_b_*	20	m
Mobile station height	*h_m_*	2	m

## Changes in Section 5. Range Versus Data Rate: Results

In paragraph 2 line 2, the sentence “Using the Free Space Path Loss model, it is found that a 2.4 GHz LoRa signal can travel up to 333 km in free space and still be received properly.” should be changed to “Using the Free Space Path Loss model, it is found that a 2.4 GHz LoRa signal can travel up to 133 km in free space and still be received properly.”

In paragraph 3 line 3, the sentences “When transmitting with a spreading factor of 12 and the lowest bandwidth (i.e., 203 kHz), the path loss equals 150.5 dB. Consequently, a maximum communication range of 107 m can be achieved. Furthermore, the highest possible data rate at that range becomes 0.595 kbit/s. At the other extreme, the highest achievable data rate of 253.91 kbit/s is possible at a range of up to 26 m.” should be changed to “When transmitting with a spreading factor of 12 and the lowest bandwidth (i.e., 203 kHz), the path loss equals 142.5 dB. Consequently, a maximum communication range of 74 m can be achieved. Furthermore, the highest possible data rate at that range becomes 0.595 kbit/s. At the other extreme, the highest achievable data rate of 253.91 kbit/s is possible at a range of up to 18 m.”

In paragraph 4 line 1, the sentence “Finally, the communication range of the urban ECC-33 path loss model varies from 25 m at the highest achievable data rate of 253.91 kbit/s to 867 m at the lowest possible data rate of 0.595 kbit/s.” should be changed to “Finally, the communication range of the urban ECC-33 path loss model varies from 3 m at the highest achievable data rate of 253.91 kbit/s to 443 m at the lowest possible data rate of 0.595 kbit/s.”

In the original article, there was a mistake in Figure 2, Figure 3 and Figure 4 as published. The X-axis data in Figure 2 should be changed from 0–340 to 0–150, the X-axis data in Figure 3 should be changed from 20–110 to 15–80, and the X-axis data in Figure 4 should be changed from 0–900 to 0–500. The corrected Figure 2 appear below.

**Figure 2:**
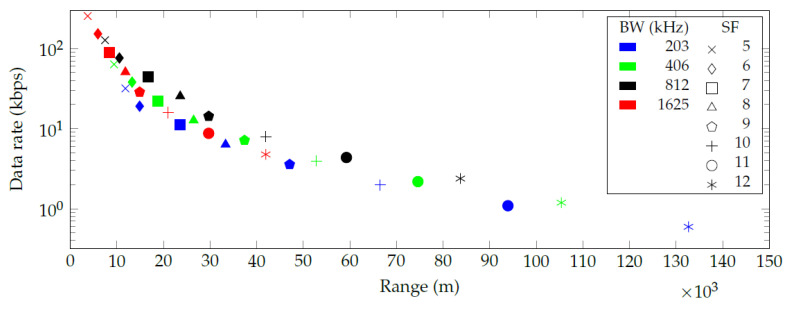
Communication range and data rate for every combination of spreading factor (SF) and bandwidth (BW) in a free space line of sight (LoS) environment.

**Figure 3:**
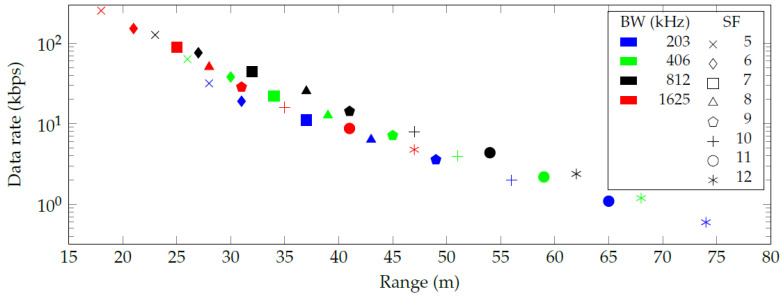
Communication range and data rate for every combination of spreading factor (SF) and bandwidth (BW) in an indoor environment.

**Figure 4:**
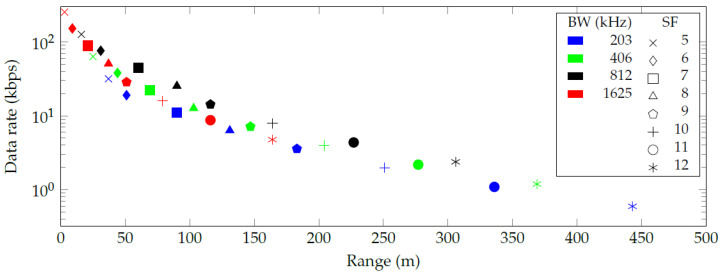
Communication range and data rate for every combination of spreading factor (SF) and bandwidth (BW) in an urban environment.

## Changes in Section 6. Discussion

In paragraph 2 line 6, the sentence “For instance, a 5 dB fade margin decreases the maximum urban range from 867 m to 576 m, while a 10 dB fade margin further decreases the range to 369 m.” should be changed to “For instance, a 5 dB fade margin decreases the maximum urban range from 443 m to 277 m, while a 10 dB fade margin further decreases the range to 164 m.” 

In paragraph 3 line 1, the sentence “The free space line-of-sight scenario resulted in a theoretical maximum range of 333 km when transmitting at the highest SF and using the lowest bandwidth.” should be changed to “The free space line-of-sight scenario resulted in a theoretical maximum range of 133 km when transmitting at the highest SF and using the lowest bandwidth.”

In paragraph 3 line 6, the sentence “For instance, the maximum range of LoRa at 868 MHz calculated with the FSPL model equals 921 km, which is almost three times the range of LoRa at 2.4 GHz.” should be changed to “For instance, the maximum range of LoRa at 868 MHz calculated with the FSPL model equals 921 km, which is almost seven times the range of LoRa at 2.4 GHz.”

In paragraph 4 line 4, the sentence “The estimated range varies from 26 m to 107 m, depending on the SF and BW.” should be changed to “The estimated range varies from 18 m to 74 m, depending on the SF and BW.”

In paragraph 6 line 4, the sentence “This data rate can be achieved if the distance between the transmitter and receiver is not greater than 9393 m, 26 m and 25 m in a free space, indoor and urban environment, respectively.” should be changed to “This data rate can be achieved if the distance between the transmitter and receiver is not greater than 3739 m, 18 m and 3 m in a free space, indoor and urban environment, respectively.”

In paragraph 7 line 4, the sentence “Thus, the outdoor range of LoRa is more than five times larger than the outdoor range of BLE 5 and more than eight times larger compared to typical IEEE 802.11 networks.” should be changed to “Thus, the outdoor range of LoRa is almost three times larger than the outdoor range of BLE 5 and more than four times larger compared to typical IEEE 802.11 networks.”

These changes have no material impact on the conclusions of our paper. The authors apologize for any inconvenience caused and state that the scientific conclusions are unaffected. 
